# The costs of developing, deploying and maintaining electronic immunisation registries in Tanzania and Zambia

**DOI:** 10.1136/bmjgh-2019-001904

**Published:** 2019-11-25

**Authors:** Mercy Mvundura, Laura Di Giorgio, Dafrossa Lymo, Francis Dien Mwansa, Bulula Ngwegwe, Laurie Werner

**Affiliations:** 1 Medical Devices and Health Technologies Group, PATH, Seattle, Washington, USA; 2 Expanded Programme on Immunizations, Ministry of Health Community Development Gender Elderly and Children, Dar es Salaam, United Republic of Tanzania; 3 National Expanded Programme on Immunization, Ministry of Health, Lusaka, Zambia; 4 Center of Digital and Data Excellence, PATH, Seattle, Washington, USA

**Keywords:** health economics, health systems evaluation, immunisation, other study design

## Abstract

**Objective:**

To determine the costs to develop, roll out and maintain electronic immunisation registries (EIRs) and a related suite of data use interventions.

**Methods:**

The Better Immunisation Data (BID) Initiative conducted the activities from 2013 to 2018 in three regions in Tanzania and one province in Zambia. The Initiative’s financial records were used to account for the financial costs of designing and developing the EIRs, BID staff time, expenditures for rolling out the EIR systems and the related suite of interventions to health facilities, and recurrent costs. Total financial costs, cost per facility and cost per child were calculated in 2018 US$.

**Findings:**

Total expenditures were ~US$4.2 million in Tanzania and US$3.6 million in Zambia. System design and development costs accounted for ~33% and 26% of the expenditures in each country, respectively, while BID staff costs accounted for 39% and 52%, respectively. Average expenditures per health facility for rolling out the EIR system were between US$709 and US$1320 for the Tanzania regions and US$2591 for Zambia. The annualised average expenditure per child was estimated to be between US$3.30 and US$3.81 for the regions in Tanzania and US$8.46 in Zambia. Expenditures per child were higher in Zambia partly because of a much smaller birth cohort compared with Tanzania.

**Conclusion:**

Other countries may benefit from the investments made and lessons learnt in Tanzania and Zambia by leveraging these now existing EIR platforms and rollout strategies, and hence may be able to implement EIRs at lower costs than reported here.

**Key questions**

**What is already known?**

- Immunisation programme in low/middle-income countries (LMICs) face challenges with data quality and use and so electronic immunisation registries (EIRs) combined with data use interventions may be a solution.
- Information on the costs of developing, deploying and rolling out the EIRs in LMICs is lacking.

**What are the new findings?**

- Evidence from Tanzania and Zambia show that the costs of developing, deploying and maintain EIRs cost <US$10 per child aged under 1 year of age, but these costs can be lower than US$5 per child in a country like Tanzania that has a large birth cohort.
- Hardware and deployment of the EIRs are the cost categories that account for a large share of these costs.

**What do the new findings imply?**

- These estimates can be used by stakeholders when planning and budgeting for similar interventions.
- Subsequent EIR development and deployment costs may be less than those reported in this study because of the ability of other LMICs to leverage these EIR systems that were developed for Tanzania and Zambia and also through leveraging learnings generated from these deployments.

## Introduction

Health programmes, including immunisation programmes, in low/middle-income countries (LMICs) face challenges resulting from poor-quality data and poor use of existing data.^1–4^ These challenges include lack of person-level health records or data formats that could enable easy access, review, analysis and use of programme data; inaccurate or uncertain target populations; incomplete data for individual patients or for the health facility; and inadequate capacity across the health system for data management and use.^1 3–5^ Because of these challenges, most health programmes, including immunisation programmes, in LMICs lack accurate information on the size of the target populations they serve and have difficulty tracking performance indicators such as the number of children not fully immunised and have difficulty in identifying children due to receive interventions and those who had missed receiving health interventions (defaulters). Some of these challenges stem from reliance on paper-based tools, registers and reports.^6^ Electronic interventions to address these challenges can be classified as development of software for (1) handling data records of individuals such as electronic health records and (2) systems associated with data collection for decision-making and information management, such as health management information systems.^7^ Most efforts in LMICs to date have been put towards the latter category, with the development of systems such as district health information software (DHIS).^7–10^ However, DHIS and similar systems lack person-level health records, and so do not address those challenges faced by health programme which require person-level data.

In an attempt to tackle these challenges, including the lack of child-level immunisation records and poor quality data for decision-making and information management, the Better Immunisation Data (BID) Initiative^11^ was implemented in Tanzania and Zambia, led by the Ministry of Health, Community Development, Gender, Elderly and Children (Tanzania) and the Ministry of Health (Zambia), in partnership with PATH, a global non-governmental organisation. The BID Initiative was launched in 2013 to lead the development and implementation of solutions to improve countries’ collection, quality and use of immunisation data. The work began with collecting common system requirements from 10 sub-Saharan African countries; these requirements represented the countries’ vision of an ideal system to track individual child vaccination schedules and elements of the immunisation supply chain. Through iterative development processes in both Tanzania and Zambia, the common requirements were modified and adapted to better fit the country contexts and users’ needs, as well as to be developed with the technology available at the time.^5 12^ Among the solutions, the most intricate to develop was the electronic immunisation registry (EIR),^13^ which now gives health workers access to child-level as well as aggregated facility-level data that can be used for decision-making to improve the effectiveness and efficiency of immunisation services. Once software development had begun in each country, a user advisory group made up of health workers from across the health system helped design a suite of interventions to address data-related challenges. Specifically, this user group gave input and feedback on how well the software was meeting the functional requirements such as registering a new child, searching for a child already registered in the system and printing reports.

When BID’s work began, there was no existing EIR system that met the requirements of each country. Part of the initial strategy of BID was to support the development of two different systems to meet diverse country needs, thus reducing the likelihood of other countries needing to invest in new software development. It is challenging to develop one system that meets diverse country needs, and so BID developed two different systems in order to provide options for countries to choose from in order to have a system that best meets their needs and country context. Hence, Tanzania and Zambia each developed their own EIR: Open Immunize (OpenIZ) in Tanzania and Open Smart Registry Platform (OpenSRP) in Zambia. Both software platforms are open source and therefore have freely available source codes for other countries interested in using similar software. The source code for both platforms is in English, but the source code for OpenIZ is also available in Swahili. These respective EIRs were then rolled out by the initiative to three regions in Tanzania and one province in Zambia.

Critical to implementing any new system is monitoring and understanding start-up and ongoing costs to ensure sustainability and scalability. For BID, this was especially important as no previous evidence existed on the costs of implementing EIRs in LMICs. Therefore, we integrated an evaluation of the total cost of ownership^14^ by quantifying the financial expenditure done by the BID initiative for developing, rolling out and maintaining the EIRs and related suite of interventions in Tanzania and Zambia. We present the total expenditure by BID in each country, the cost per health facility of rolling out the interventions, and the annualised cost per child in the expanded programme on immunisation (EPI) target population.

## Methods

### Description of the intervention

Before implementation of the EIRs, health workers in both countries were using paper-based immunisation registers, tools and reports. The EIRs were implemented and designed for health workers to electronically register and track children through their vaccine schedules and to automate reporting. At implementation start-up, tablets preloaded with EIR software and barcode/quick response code scanners were provided to health facilities as the core tools for interfacing with the EIR system.

When a child first presents at a health facility, the child is registered into the EIR with a unique identification number. A label containing a barcode or quick response code with this number is then placed on the child’s health card. The child’s record can then be accessed at any facility the child visits. If the caregiver does not have the child’s health card at the next visit, staff can search the registry using the child’s name, caregiver’s name, village or unique identification number. The child record in the EIR includes the child’s demographic information such as their gender and date of birth and also vaccination information such as vaccination schedule and records including vaccines they have received in the past and those they are due for. In addition, information on the child weight, vitamin A receipt, nutrition and deworming status are included, as these services are provided together with immunisation services. Caregiver information such as their name, village and contact information are also included in the electronic record and can be used contacting the caregiver when the child is due for their next vaccination or following up if the child has missed an appointment. Both countries’ EIRs also contain simple reports that inform health workers when a child is due for the next vaccination and whether he or she has missed a scheduled appointment. The health worker can use this information to follow-up with caregivers. The EIRs also contain an inventory management component that can be used to show when a facility is running low on vaccine stock or supplies. The EIRs developed through the Initiative focused mainly on immunisation; however, given that other health services are provided at the same time as vaccinations, these data were also captured. The EIR systems are both web-based and tablet-based, in on and offline functions. The data in the EIR mirrors the monthly child health and immunisation reports the health workers completed. The systems used in both countries are interoperable with DHIS-2 in both countries, and the governments are in the process of adjusting policy to receive the reporting information into the DHIS-2 electronically. Additional details on the functionality of the EIRs are provided in previous publications.^15^

This study’s analysis focuses on the development and rollout costs of the BID Initiative: table 1 shows the regions and province included in the analysis, the number of health facilities to which the EIRs were deployed and the EPI target populations. Rollout to a fourth region in Tanzania and portions of another province in Zambia were conducted by other partners; those financial records were neither accessed nor included.

**Table 1:** Regions/province included in the analysis and number of facilities and children

Country	Region/ province	No of districts	No of health facilities with EIR software deployed	No of children aged 0–12 months	EPI annual target population for the country (2017)^24^
Tanzania	Arusha	7	285	133 571*	2 083 000
Tanzania	Tanga	11	327	96 950*	
Tanzania	Kilimanjaro	7	312	88 233*	
Zambia	Southern	13	294	78 071†	618 000

*Census data on EPI target population.

†Data from Southern Province EIR.

EIR, electronic immunisation registry; EPI, expanded programme on immunisation.

### Development and rollout

In 2014, work began in Tanzania on development of an EIR. This system was initially deployed in Arusha Region but was later shelved due to challenges that arose during deployment. A second EIR system was developed on the OpenIZ platform, which is the EIR included in this costing analysis and currently in use in Arusha, Tanga and Kilimanjaro regions (and applicable to all regions in the country). In Zambia, initial work on the EIR platform DHIS eTracker was never deployed. In 2016, a different partner developed an EIR system for Zambia on the OpenSRP platform. This is now in use in Southern Province and is applicable to other provinces in the country. Our study includes the costs for developing the initial EIRs which were later shelved; these ‘learning costs’ are included as the upper-bound estimates of the study.

The Initiative intended to use data capture software to back enter vaccination records for children aged <9 months from the paper registers into the EIRs; otherwise, health workers would have to back enter the complete record of each child at the child’s first encounter with the new system. However, the data capture software did not work as expected, and there was no suitable alternative other than manually entering the records. This manual back entry was done by contracted staff in each country until the initial cohorts of children were registered in the EIRs.

Deployment of the EIR was done through a series of on-the-job trainings for health workers. A tablet, charger and barcode/quick response code scanner were provided to each health facility, with a few facilities that served larger populations receiving two tablets and scanners. During the visits, health workers in the immunisation programme were introduced and trained on the EIR and related suite of interventions, which included targeted supportive supervision for facilities, peer support networks, data use guides and posters, and simplified and visualised reporting. This training was done in the context of the clinical care workflows and procedures and aimed to ensure that health workers were equipped to strengthen data collection, improve the quality of data available and increase the use of the data for decision-making.^12^ Each visit typically lasted about 6 hours with an average of four visits per facility. More visits were conducted initially and then streamlined as the deployment gained momentum and greater efficiency. The learnings from each rollout was taken into account and the rollout strategy was refined to increase likelihood for sustainability and scale-up. For example, subsequent rollouts included district level data use mentors who were integrated as staff at the district immunisation office and were available to provide monitoring and supportive supervision for longer periods of time.^16^ In Arusha Region, Tanzania, the visits were done by PATH staff. In Tanga and Kilimanjaro regions, implementation was transitioned to district-level government staff. With each subsequent deployment, the strategy was adjusted and improved based on learnings. In Zambia, PATH staff conducted the on-the-job trainings for health workers in partnership with government staff, with one additional off-site training for health workers from across the province. Additional details on how the BID interventions were rolled out and learnings from these rollouts are available in previous publications.^15 16^

### Costing methods

This analysis focuses on the financial expenditures incurred by the BID Initiative in Tanzania and Zambia for developing the EIRs, deploying them and the related suite of interventions and maintaining the systems. This analysis does not include economic costs. By focusing on the financial costs, we present actually monetary outlays incurred for developing and deploying the EIRs. Financial costs are most relevant for budgeting and planning and we present these to provide an accounting of the expenditures that were spent on developing, deploying and maintaining EIRs. The data were obtained from BID’s financial records and tracked over the period 2013–2018. We focused on BID’s financial costs hence the opportunity costs for government staff for time to participate in meetings, be involved in training (on the job or offsite) and use of existing government resources such as vehicles to attend the meetings were not captured. These costs would have been included if this analysis was focused on economic costs which take into account the opportunity costs of using existing resources. The financial expenditures for government staff that were accounted for in this analysis were those covered by BID, such as per diems for government staff to attend the meetings and fuel and transportation allowances when government vehicles were used. The analysis focuses on financial expenditure and so the full costs for system development and hardware procurement are included in the analysis. As a result, capital depreciation costs are not included as this would be double counting of costs. Any costs for system maintenance and upgrades that were incurred during the 5-year project period are also accounted for and included. The analysis also includes the costs that were borne in each country and excludes project costs for PATH staff based in the headquarters office or in-country that were related to administration and research. Table 2 shows the expenditures included in this study and how they were classified. In this study, we use the terms cost and expenditure interchangeably.

**Table 2:** Categories of costs included in the financial expenditure analysis

Financial cost category	Cost description
**System design and development**	
System design and development (EIR which is being used)	Design and development of the EIR in each country; includes testing the EIRs.
	
Learning costs	Design and development of first version of EIRs in each country which were later shelved.
**Other costs**	
Back entry costs	Costs to enter previous immunisation records of each child from the paper immunisation registers into the electronic immunisation registry.
Peer learning and printing of guidelines (Tanzania only)	Printing of guidelines and a visit to Zambia for peer learning exchange.
**Labour costs**	
BID initiative staff	BID initiative staff time, including in-country staff time for all project-related activities such as system testing, deployment, supervision and management of the project.
**Intervention rollout costs by region/province**	
Hardware*	Tablets and covers, chargers and quick response code/barcode scanners for health facilities.
Meetings	Meetings with government officials to get their buy-in and to plan for implementation in their region/province; includes meetings associated with developing the rollout strategy for the region/province.
Training	Training of staff responsible for rolling out the interventions to the health facilities.
Deployment	Per diems, lodgings and transport associated with deployment of the EIR to the health facilities and district immunisation offices. Transport includes vehicles purchased (one for each country) and expenditures for fuel and maintenance of these vehicles; also includes hiring other vehicles used for the deployment.
**Recurrent costs by region/province**	
Internet connectivity	Access to internet for uploading data and transferring data to higher levels.
Data hosting	Server for EIR data.
Supportive supervision	Per diems and transport costs for BID initiative staff or Ministry of Health staff to provide supportive supervision to health facilities after deployment of interventions.
Printing	Printing of barcodes used on immunisation cards.

*Includes equipment replacement at a rate of 5% as noted over the project period, which is included in the equipment procurement costs.

BID, Better Immunisation Data; EIR, electronic immunisation registry.

In Tanzania, some expenditures were not reported separately by region. These included costs for data hosting, internet, vehicle hire and printing. As a consequence, costs were allocated to each region based on the relative size of the EPI target population.

We present the total expenditures for BID related to the EIRs and associated interventions. We also present the average costs per health facility for rolling out the EIR systems which were calculated by dividing the total regional/provincial costs for rolling out the EIR by the number of health facilities in each region/province. In addition, we calculated the cost per child in the EPI target population. To do this, we annualised the costs in each of the categories included in table 1 (except for recurrent costs which were not annualised) over a 3-year period. We then divided the annualised costs by the annual birth cohort served by the nation or region/province, depending on the reach of the expenditure. Since the EIRs were designed and developed for use nationwide, we calculated the cost per child for system development by dividing the annualised system design and development expenditures by the size of the country’s annual birth cohort. We also allocated BID staff labour costs between national and regional/provincial EPI cohorts and estimated that ~60% of the BID labour costs were attributable to the national-level work (design and development of the tools; national-level stakeholder meetings) and 40% to regional-/provincial-level work (direct deployment of the interventions). For the region-/province-specific cost per child, we used the regional/provincial population EPI target population. The calculation is illustrated below:

Financial expenditure for the EIR systems and related suite of interventions per child =

*​(annualised design and development costs for system in use / national EPI target population) +*

*​(annualised learning costs for design and development / national EPI target population) +*

*​(annualised peer learning costs / national EPI target population) +*

*​(annualised back entry costs / region or province EPI target population) +*

​(annualised BID labor costs*0.6 / national EPI target population) +

​(annualised BID labor costs*0.4 / region or province EPI target population) +

*​(annualised region or province intervention rollout costs + annual region or province recurrent costs) / region or province EPI target population.*

All costs are presented in 2018 US$.

Ethics clearance was not required for this study because the data for the analysis were obtained from project financial records.

### Patient and public involvement

This research was done without patient or public involvement. It was not appropriate or possible to involve patients or the public in the design, or conduct, or reporting, or dissemination of our research as the study focused on analysis of expenditure data.

## Results

Table 3 shows that the total in-country expenditures for design and development of the EIRs, regional deployment of the interventions and recurrent costs were ~US$4.2 million in Tanzania and US$3.6 million in Zambia. System design and development costs, excluding learning costs, were ~US$868 000 (21% of the total financial expenditures) in Tanzania and US$487 000 (14% of the total expenditures) in Zambia. Including the learning costs (US$528 000 in Tanzania; US$427 000 Zambia) would increase the percentage of costs for system design and development to 33% and 26%, respectively. Labour costs for BID staff accounted for 39% of total project expenditure in Tanzania; 52% in Zambia. It is important to note that BID staff rolled out the interventions in Arusha Region (Tanzania) and Southern Province (Zambia), while in Kilimanjaro and Tanga regions (Tanzania), government staff led the implementation with support from BID.

**Table 3:** Total financial expenditures for development and rollout of the EIRs with the related suite of interventions for the period 2013–2018

	Tanzania	Zambia
**System design and development**		
System design and development costs of electronic immunisation registry (in use)	US$867 851	US$486 965
Learning costs (electronic immunisation registry which was shelved)	US$527 644	US$427 407
**Other costs**		
Back entry costs	US$84 441	US$21 086
Peer learning and printing of guidelines (Tanzania only)	US$6242	–
**Labour costs**		
BID initiative staff	US$1 648 484	US$1 851 105

Region / province-specific costs				
	Arusha	Tanga	Kilimanjaro	Southern
**Rollout costs**				
Hardware	US$187 232	US$158 588	US$93 289	US$254 424
Meetings	US$8728	US$9783	US$7097	US$32 470
Training	US$33 232	US$20 686	US$17 148	US$29 368
Deployment	US$146 701	US$162 353	US$103 617	US$445 655
**Annual recurrent costs**				
Internet connectivity	US$16 930	US$19 807	US$16 301	US$20 007
Data hosting	US$9086	US$10 630	US$8308	US$2061*
Supportive supervision	†	US$6178	US$8786	†
Printing	US$4702	US$5501	US$4300	US$2926
Total country costs over the project period	US$4 193 647			US$3 573 474

*Financial cost only for 3 months. Hence, annual costs would be ~US$8000.

†Not tracked separately in the financial records but included in the rollout costs.

BID, Better Immunisation Data.

When looking at the region-/province-level expenditures for rollout, we observed that expenditures for hardware and for deploying the interventions to health facilities and districts was the largest share of the expenditure. Total expenditures for hardware ranged from about US$93 000 to US$187 000 per region in Tanzania (table 3) and about US$254 000 in Southern Province. The total expenditure for deploying the EIR system to health facilities and district immunisation offices ranged from US$103 617 to US$162 353 in Tanzania and was US$445 655 in Southern Province, Zambia.

Recurrent costs accounted for 2.6% of the initiative’s total expenditures in Tanzania and 0.7% in Zambia (table 3). Of these costs, internet connectivity represented the largest share and ranged from US$16 000 to US$20 000 per year per region/province; data hosting ranged from US$8000 to US$11 000 per region in Tanzania. Total annual recurrent expenditures ranged from US$31 000 to US$42 000 for the Tanzania regions and extrapolated to around US$31 000 for Southern Province, Zambia.

When looking at the expenses per health facility (including hardware to district offices) for Tanzania, these expenditures averaged US$299 to US$657, while for Zambia the average expenditure was US$865 (table 4). The average expenditure for deploying the EIR system ranged from US$332 to US$515 per health facility in Tanzania; US$1516 per health facility in Zambia (table 4). The total average expenditure for rolling out the EIR system was between US$709 and US$1320 for Tanzania and US$2591 for Zambia.

**Table 4:** Average expenditure per health facility for the electronic immunisation registry rollout

	Tanzania			Zambia
	Arusha region	Tanga region	Kilimanjaro region	Southern province
**Rollout costs**				
Hardware	US$657	US$485	US$299	US$865
Meetings	US$31	US$30	US$23	US$110
Training	US$117	US$63	US$55	US$100
Deployment	US$515	US$496	US$332	US$1516
Total average expenditure per health facility	US$1320	US$1074	US$709	US$2591

**Figure 1:**
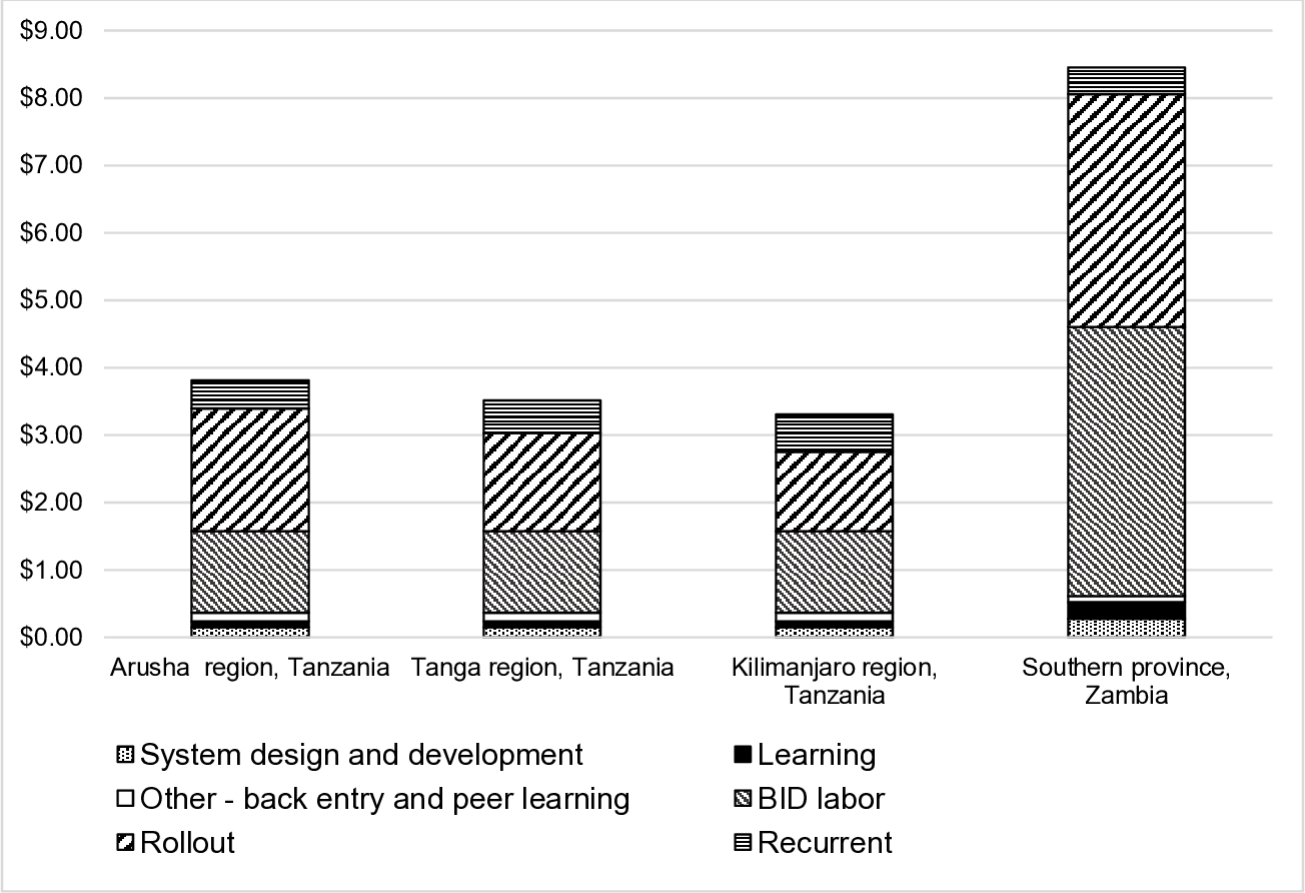
Annualised expenditures per child for the development and rollout of an electronic immunisation registry in Tanzania and Zambia. BID, better immunisation data.

Figure 1 shows that the total annualised expenditure per child in the EPI target population ranged from US$3.30 to US$3.81 for the three regions in Tanzania when including learning costs; US$3.22 to US$3.72 when excluding learning costs. The expenditures for rolling out the EIR system were estimated at between US$1.17 and US$1.82 per child, or 36% and 48% of the annualised cost per child. Rollout costs declined with each subsequent rollout in Tanzania as the strategy was adjusted based on learnings. For Zambia, the annualised expenditure per child was much higher—estimated at US$8.46 when including learning costs; US$8.21 when excluding. The expenditures for rolling out the EIR system were estimated at US$3.45 per child, or 41% of the costs. Overall the costs were higher in Southern Province because of the compressed timeline, which raised implementation and vehicle costs.

## Discussion

Our evaluation shows that the total costs for developing, rolling out and maintaining the BID interventions were US$4.2 million and US$3.5 million in Tanzania and Zambia, respectively, over the 5-year period. We estimated an annualised cost per child ranging from US$3.30 to US$3.81 in the three regions in Tanzania and US$8.46 in the one province in Zambia. Our literature search did not find any previous studies that had evaluated costs of EIRs in LMICs; limited evidence on the costs of EIRs is from high-income countries and thus hardly comparable to settings in sub-Saharan Africa. For example, a study conducted in Massachusetts, USA, in 2002 found that the cost per child for an immunisation registry was US$8.46 per child under 23 years of age and US$15.51 per child when including only children under 8 years of age (adjusted to 2018 US$).^17^ A study conducted in California, USA^18^ found that the cost to develop an immunisation registry was about $388 000 adjusted to 2018 US$ (US$250 000 in 1998 US$). We also conducted literature searches to find information on the expenditure on other interventions to improve data such as through implementing DHIS-2. We did not find any costing estimates related to DHIS-2, which would have been a close comparator, though also not providing the whole solution set as the intervention we are evaluating. Therefore, there is a dearth of evidence on the costs of implementing interventions to improve data use and quality.

Our study found that hardware and deployment costs were the two largest expenditures associated with rolling out the interventions to the health facilities. This is not unexpected given the equipment that has to be procured for each health facility and also the per diem, lodging and transport expenditures to travel to each health facility to train the healthcare workers on using the new system.

We found that the rollout costs for Arusha region were higher than for the other two regions in Tanzania because some facility visits in Arusha region had to be repeated after the EIR system was redesigned in order to train health workers on the new EIR system. However, each subsequent rollout in Tanzania had lower costs because implementing the learnings gained from previous rollouts reduced the costs of conducting the deployment of the interventions in each subsequent region.

While we present the data for Tanzania and Zambia together, the aim of this evaluation was not to compare results between the two countries due to significant differences in country context. For example, EIR development costs in Tanzania were higher in Tanzania than in Zambia because Tanzania was the first country to engage in the software development activities and more activities were conducted in Tanzania than in Zambia. Some of these include the landscaping analysis done during the design phase work to ensure interoperability with existing systems. The learnings from the initial Tanzania experience informed the Zambia development process, where the team identified challenges earlier and more swiftly to inform the change to a second software at an earlier stage. In Zambia, the intervention rollout costs were higher due to delays in system development which required the team to adopt relatively more expensive strategies to complete the rollout within the initiative’s funding time frame. In addition, the strategies used to roll out the interventions differed in the two countries, with Tanzania able to use more government staff time for implementation in the Tanga and Kilimanjaro regions compared with Zambia, where the government could provide only limited implementation support in Southern Province. Hardware costs were also higher in Zambia because the per unit costs for tablets was higher in Zambia than in Tanzania. Some of these differences reflect differences in local procurement costs for electronics. Our results also show that the cost per child in Zambia was much higher than in Tanzania despite similar total financial expenditures. One reason is that Zambia had a much smaller national birth cohort than Tanzania (about a third of the size), hence, the costs in Tanzania were spread over a larger group of beneficiaries. In general, because of economies of scale, the cost per child would be lower in larger countries because these costs are spread over a larger EPI population. Furthermore, the Zambia rollout did not benefit from expanding to other provinces and capitalising on the lessons learnt in Southern Province, which would presumably reduce the average cost of rolling out the interventions as it did in Tanzania. It will be important for the programme to continue to share the costs of deployment to additional regions or provinces and facilities to enable comparison with the costs we present here and also to inform decision-making and budgeting. Tanzania has continued to rollout the EIR to additional regions after the BID initiative ended. The government of Tanzania is now scaling the EIR nationally with their Gavi Health System Strengthening funds, and to date (October 2019) the EIR is currently in nine regions with plans to scale the rest of the country in 2020. The government of Zambia is also using their Gavi funding to continue work in Southern Province and has provisional approval on funding to expand to Western Province.

The following considerations should be kept in mind when using these findings as a benchmark. First, the expenditures we report included the actual software development costs in each country, which is a significant expense that other countries may not have to incur, as they can build from these EIR systems that are available now. Second, learning costs associated with the EIRs that were shelved increased system development and rollout costs for Tanzania and Zambia; again, other countries need not go through these same learning processes as the intent is that they can build off of the either of the software systems developed for Tanzania or Zambia. Third, BID staff labour costs were a significant share of the expenditures; costs may be lower if government staff conduct some of the activities undertaken by BID. For these reasons, the costs we report here may be the upper-bound costs for EIR system development and rollout in LMICs. It should also be noted that recurrent costs may shift as governments take over the running of EIR systems, depending on how they choose to host the data, how they conduct the maintenance and management of the system and what level of ongoing training and support they choose to provide to users. Additionally, as governments scale up EIRs to other regions, financial expenditures may change as they use existing resources or choose different strategies to implement and deploy interventions. Although we found that recurrent costs were a small share of the expenditures, governments may (or may not) be able to negotiate reduced rates for costs such as internet connectivity and data hosting. Nevertheless, our study can be used to estimate future expenditures for EIR development and deployment in LMICs. Finally, the expenditures presented here may seem a relatively large share of the country’s immunisation spending. Tanzania’s total expenditure for the routine immunisation programme was ~US$73 million in 2017 while for Zambia it was US$38 million.^19 20^ However, it should be noted that a sizeable amount of funding that LMICs have been using to improve data systems have come as additional funding from donors such as Gavi through funding mechanisms such as the health system strengthening funds. In 2019, Tanzania was approved by Gavi to receive US$7.4 million over a 5-year period million to support efforts to improve immunisation data management and utilisation at all levels.^21^ In 2017, Zambia was approved by Gavi also to receive ~US$2 million over a 3-year period to support efforts to improve the collection and utilisation of HMIS data at all levels of the healthcare system with special focus on district and lower levels.^22 23^

This analysis had several limitations. First, we included the financial expenditures only of the BID initiative; because this analysis focuses on financial rather than economic costs we did not quantify the opportunity costs for government employees’ time spent in trainings or the economic costs of using existing government resources for deploying the interventions. Second, in this study, we only present expenditures and not the benefits of the EIRs; however, these benefits were evaluated and will be disseminated through other publications by the BID initiative research team. Last, electronic registries are an important source of more reliable denominator data, yet we present our cost-per-child estimates using EPI target population data. To the extent that the EPI target populations are underestimated, we have overestimated the cost per child and vice versa.

## Conclusion

Our study may be the first to provide the total costs of ownership for EIR development and rollout at scale in a province or region in Africa. Stakeholders can use this evidence to benchmark expenditures for future investments. We anticipate that other countries will benefit from the investments made and lessons learnt in Tanzania and Zambia by leveraging these now existing EIR platforms and rollout strategies, and hence may be able to implement EIRs at lower costs than reported here.
